# *Aspidosperma* species as sources of anti-malarials: uleine is the major anti-malarial indole alkaloid from *Aspidosperma parvifolium* (Apocynaceae)

**DOI:** 10.1186/s12936-015-0997-4

**Published:** 2015-12-10

**Authors:** Maria Fâni Dolabela, Marinete Marins Póvoa, Geraldo Célio Brandão, Fabíola Dutra Rocha, Luciana Ferreira Soares, Renata Cristina de Paula, Alaíde Braga de Oliveira

**Affiliations:** Programa de Pós-Graduação em Ciências Farmacêuticas, Instituto de Ciências da Saúde, Universidade Federal do Pará, Belém, PA 66075-110 Brazil; Laboratório de Malária, Seção de Parasitologia, Instituto Evandro Chagas, Br-316, Km 7, s/n, B. Levilândia, Ananindeua, PA CEP 67030-000 Brazil; Escola de Farmácia, Universidade Federal de Ouro Preto, Ouro Preto, MG 35400-000 Brazil; Faculdade de Farmácia e Bioquímica, Universidade Federal de Juiz de Fora, Juiz de Fora, MG 38036-900 Brazil; Faculdade de Farmácia, Universidade Federal de Minas Gerais, Av. Antônio Carlos, 6627, Pampulha, Belo Horizonte, MG 31270-901 Brazil

**Keywords:** Anti-malarial activity, *Plasmodium falciparum*, Alkaloids, Uleine, Apocynaceae, *Aspidosperma parvifolium*

## Abstract

**Background:**

Several species of the genus *Aspidosperma* (Apocynaceae) are used for the treatment of human malaria in Brazil and other meso- and South American countries.

**Methods:**

Ethanol extract from *Aspidosperma parvifolium* trunk bark was submitted to acid–base extractions leading to alkaloid and neutral fractions. The alkaloid fraction was chromatographed over a silica gel column. Ethanol extract, fractions and uleine were analysed by HPLC–DAD, UPLC-ESI–MS/MS and HPLC-ESI-MicroTOF-MS. The anti-malarial activity was assayed against resistant and sensitive chloroquine *Plasmodium falciparum* strains by microscopic, [^3^H]-hypoxanthine incorporation and HRPII techniques. Cytotoxicity (CC_50_) was evaluated against Vero and HepG2 cell lines by the MTT technique; selectivity indexes (SI = CC_50_/IC_50_) were calculated.

**Results:**

The major peak in the HPLC–DAD chromatograms of the ethanol extract, alkaloid and neutral fractions suggested the presence of uleine that was isolated from the alkaloid fraction by column chromatography and was characterized by spectroscopic methods. A total of 15 alkaloids, besides uleine, were identified in the alkaloid fraction by UPLC-DAD-ESI–MS/MS and HPLC-ESI-MicroTOF-MS. The ethanol extract from *Aspidosperma parvifolium* and the neutral fraction were moderately active against *P. falciparum* strains. The alkaloid fraction and uleine disclosed high anti-malarial activity against chloroquine-resistant *P. falciparum* strain (IC_50_ < 1 µg/mL). The ethanol extract, neutral fraction and uleine showed low cytotoxicity against Vero and HepG2 cell lines (CC_50_ > 300 µg/mL). The alkaloid fraction showed moderate cytotoxicity to HepG2 cell line (CC_50_ = 74.4 µg/mL). High SI values (>10) were determined for all samples.

**Conclusion:**

Ethanol extract from *Aspidosperma parvifolium* trunk bark afforded uleine that is the major constituent of the alkaloid fraction and disclosed a good in vitro anti-malarial activity. Moreover, 15 other indole alkaloids have been identified along with uleine.

## Background

Globally, 3.3 billion people are at risk of malaria, a disease endemic to more than 100 countries. According to latest estimates, 198 million cases of malaria and 584,000 deaths occurred in 2013; 90 % of all malaria deaths occurred in Africa and 78 % of all deaths in children under 5 years old [1]. The extensive use of anti-malarial drugs has imposed a high selective pressure on parasites, leading to the emergence of drug resistance, particularly in *Plasmodium falciparum* [2]. *Plasmodium falciparum* resistance to artemisinins has been detected in four countries of the Southeast Asia region. Artemisinin derivatives comprise the therapeutic scheme recommended for malaria treatment [3]. This points to the urgency for the development of new anti-malarial drugs. Plants continue to be a valuable source of bioactive compounds and investigation of traditional medicines used to treat malaria in endemic countries has afforded useful anti-malarial drugs, such as quinine, artemisinins and atovaquone [4–6].

Representatives of the genus *Aspidosperma* (family Apocynaceae, *tribo* Plumeriae) are found exclusively in the New World, from Mexico to Argentina [7], and several species have been traditionally used for the treatment of human malaria [8–11]. A recent review of the traditional use and anti-malarial activity of *Aspidosperma* species revealed several scientific bibliographical references on the use of 24 species to treat malaria/fevers, including *Aspidosperma parvifolium*, and to 19 *Aspidosperma* species that have had their extracts and/or alkaloids evaluated for in vitro and/or in vivo anti-malarial activity showing positive results. Only 20, out of more than 200 known indole alkaloids from *Aspidosperma* species have been assayed for anti-malarial activity, and variable levels of parasite inhibition have been reported [12].

*Aspidosperma parvifolium* is a tree of 10–15 m height, with a trunk diameter of 40–60 cm. It reaches approximately 4 m height in about 2 years when growing in the field and it is valuable as timber [13]. Its popular names in Brazil are *peroba, pau*-*pereira, guatambu, guatambu*-*rosa, guatambu branco, guatambu*-*oliva, guatambu*-*marfim, amarelão* [13, 14].

Uleine, 3-*epi*-uleine, apparicine, *N*-demethyluleine, lupeol, and stigmasterol were previously isolated from the trunk bark of *Aspidosperma parvifolium* [15]. Uleine stimulated a maximum nitric oxide production in the concentrations of 0.1 µg/mL (20.9 ± 1.4 µM) and 1 µg/mL (41.1 ± 0.2 µM) [16] and exhibited high level of acetylcholinesterase inhibition [17]. Uleine was evaluated for cytotoxicity against 65 cancer cell lines panel and it was inactive in all of them [18].

The present paper reports on the anti-malarial activity of *Aspidosperma parvifolium* against chloroquine-resistant (W2) and sensitive (3D7) *P. falciparum* strains. Moreover, phytochemical studies and cytotoxicity evaluations are also described.

## Methods

### Plant material, extraction and phytochemical studies

Trunk bark of *Aspidosperma parvifolium* was collected in the municipality of Paracatu, Minas Gerais, Brazil. A dried specimen was deposited in the Herbarium of the Universidade Federal de Minas Gerais (UFMG), Belo Horizonte, Minas Gerais, Brazil (voucher number BHCB60345). Plant bark was dried in an oven with circulating air at 40 °C and milled. Powdered plant bark (2.5 kg) was extracted by percolation with ethanol 96 °GL. The extractive solution was concentrated in a rotary evaporator to give the crude ethanol extract (240 g, 9.6 %). An aliquot of ethanol extract (55.2 g) was suspended in aq. HCl 1 N (500 mL) and was extracted with dichloromethane (5 × 200 mL). The organic solvent was removed in a rotary evaporator affording the neutral fraction (8.7 g). The aqueous solution was made alkaline (pH 10) with conc. ammonium hydroxide, followed by extraction with dichloromethane (5 × 200 mL). Solvent concentration in a rotary evaporator afforded the alkaloid fraction (3.8 g). Part of the alkaloid fraction (1.0 g) was chromatographed in a silica gel column eluted with solvents and mixtures of solvents of increasing polarities. Fractions eluted with ethyl acetate–methanol were combined (507 mg) and chromatographed on a silica gel column affording uleine as white crystals (49 mg).

The ethanol extract, alkaloid fraction, neutral fraction, and uleine were analysed by high performance liquid chromatography with diode array detector (HPLC–DAD, Waters mod. 2695, USA), C-18 reverse phase column (5 μm, 125 × 45 mm, LiChrocart 125-4, Merck, Germany), at 40 °C, flow rate of 1 mL/min, wavelengths scanning from 220 to 400 nm. The mobile phases were a buffer solution pH 5.0 of acetic acid with 0.2 %, triethylamine (A) and acetonitrile (B) in the following programme of elution: t = 0 min, A = 90 %; t = 10 min, A = 80 %; t = 30 min, A = 50 %; t = 35 min, A = 90 %. After 35 min acetonitrile 95 % was used for 10 min to wash the column.

Analyses by ultra-performance liquid chromatography coupled to UV and mass spectrometry by electron spray ionization (UPLC-DAD-ESI-MSMS) were performed in an Acquity H-Class Core System^®^ Waters equipment; the positive mode was used for ESI-MSMS, with capillary voltage of 3.5 eV, the cone 60 eV, CSH130 C-18 column (particles of 1.7 μm, 50 × 3 mm), flow of 0.3 mL/min, temperature of 40 °C and UV detection between 220 and 400 nm. As mobile phase, a linear gradient was used, in which the initial time contained aqueous solution of formic acid 0.1 % (A) and acetonitrile with formic acid 0.1 % (B), in 10 min 5 % of A and 95 % B. Operating parameters of the mass spectrometer were capillary temperature 250 °C; spray needle voltage set at 3.50 kV; ES capillary voltage +3 and −47 V for positive and negative polarity, respectively; tube lens offset 0 and −25 V for positive and negative polarity, respectively. Nitrogen was used as a sheath gas with a flow of 50 arbitrary units. Mass analyses were carried out in full-scan mode from 100 to 1500 Da, in both positive and negative mode. UV spectra (200–400 nm) from the main peaks were registered on line.

Uleine was identified on the basis of spectroscopic methods (Ultraviolet/UV, Infra-red/IR, ^1^H and ^13^CNMR (Hydrogen and Carbon-13 Nuclear Magnetic Resonance) and direct comparison with authentic sample previously isolated at UFMG [15].

### Anti-malarial activity

*Plasmodium falciparum* W2 (resistant to chloroquine) and 3D7 (sensitive to chloroquine) strains were maintained in continuous culture in human erythrocytes (blood group O+) in RPMI medium supplemented with 10 % human plasma (complete medium) [19]. Synchronization of parasites was achieved by sorbitol treatment [20] and parasitaemia was microscopically determined in Giemsa-stained smears.

### In vitro growth inhibition of *Plasmodium falciparum* (W2 and 3D7) by the microscopic method

Parasites in the trophozoite stage were used (parasitaemia of 1 % and haematocrit of 0.5 %) and different concentrations of test samples (≤100 μg/mL). Chloroquine was used as a standard anti-malarial drug. After 72 h of incubation under CO_2_, stained with Giemsa were prepared.

The antiparasitic effects of the extracts/fractions/compounds were measured by the per cent inhibition of parasite growth in relation to the control (parasites cultivated in drug-free medium) [21]. The 50 % inhibitory concentrations (IC_50_), compared to the drug-free control responses, were estimated by linear interpolation [22]. Each experiment was performed in triplicate and repeated three times. The blood smears were read in a double-blind manner. The IC_50_ was determined by means of linear regression curve. The following criteria being adopted: IC_50_ 10 μg/mL, good activity; IC_50_ >100 μg/mL, inactive; IC_50_ of 10–50 μg/mL, moderate activity; and, IC_50_ of 50–100 μg/mL, low activity [23].

### In vitro growth inhibition of *Plasmodium falciparum* (W2 and 3D7) by [^3^H]-hypoxanthine incorporation

Trophozoite stage parasites (1 % parasitaemia and 0.5 % haematocrit), different concentrations of test samples (≤100 μg/mL) and chloroquine as standard anti-malarial drug were used in the assays. After 24 h incubation period, 25 µL of medium containing [^3^H]-hypoxanthine (0.5 µCi/well) (PerkinElmer) was added per well, followed by another 18 h incubation at 37 °C [24]. The cells were harvested (Cell Harvester PerkinElmer) on glass fibre filters (Filtermat A, PerkinElmer) then placed onto sample bags (PerkinElmer) and immersed in scintillation fluid (UltimaGold, PerkinElmer). Radioactive emission was counted in a 1450 Microbeta TriLux Microplate Scintilation and Luminescence Counter (PerkinELmer). The inhibition of parasite growth was evaluated from the levels of [3H]-hypoxanthine incorporation, i.e., IC_50_ values were evaluated by comparing the incorporation in drug-free control cultures and estimated by linear interpolation [25] using curve fitting software (Microcal Origin software 8.5). All experiments were performed three times, and each sample was tested in triplicate.

### In vitro growth inhibition of *Plasmodium falciparum* (W2) by HRPII method

HRPII assays were performed as previously described [26]. Briefly, cultures of *P. falciparum* (1.5 % haematocrit, 0.05 % parasitaemia) were placed in 96-well microplates with the extracts/fractions/compounds and controls at different concentrations, and were incubated for 48 h at 37 °C. After 24 h incubation, the content of six wells corresponding to medium and no test sample controls were harvested and frozen in microtubes, to allow subtracting the average value obtained from these wells from the other wells for excluding the background value (production of HRP2 during the first 24 h of incubation). After a total of 48 h incubation, the plates were frozen and thawed twice for total erythrocyte lyses and 100 µL/well of the material was placed in another plate for the ELISA test. This plate was pre-coated overnight at 4 °C with 1 mg/ml of the primary antibody anti-HRPII (MPFM-55A ICLLABs) and then the content was discarded, replaced by the blocking solution (PBS/BSA 2 % 200 µl/well), incubated for 2 h, and finally the content was discarded. The haemolyzed cultures were transferred to the ELISA pre-coated plate, incubated (1 h, room temperature), discarded, incubated for 1 h with 0.05 mg/mL of the secondary antibody (MPFG55P-ICLLAB; 100 µL/well), then incubated with 100 µL/well of TMB chromogen (15 min at room temperature) in the dark. The reaction was stopped with 50 mL/L of 1 M sulfuric acid and the absorbance was read at 450 nm in a spectrophotometer (Infinite^®^200 PRO, Tecan). The results were evaluated with the software MicrocalOrigin 8.5 for determination of the dose–response curves plotted with sigmoidal fit. The 50 % inhibitory concentration growth of the parasites (IC_50_) was determined by comparison with controls with standard drug and without drugs.

### Cytotoxicity assays

Cell viability was determined by the MTT [3- (4,5- dimethyltrazol-2-yl)-2,5-diphenyl tetrazolium bromide] method according to Mosman [27]. HepG2 A16 cells and Vero (4 × 10^5^ cells/0.1 mL) were grown in RPMI-1640 (Roswell Park Memorial Institute 1640) medium (Sigma Aldrich^®^, USA), supplemented with 5 % of fetal calf serum, kept in a 5 % CO_2_ atmosphere at 37 °C. The ethanol extract from *Aspidosperma parvifolium*, fractions and uleine were solubilized in RPMI-1640 and dimethyl sulfoxide (0.02 %, v/v). After 24 h, the solution was added at different concentrations (in μg/mL: 1, 10, 100, 1000), followed by 24 h of further incubation. The MTT (2.0 mg/mL) was added, followed by incubation at 37 °C in an atmosphere of 5 % CO_2_ for 4 h. Dimethyl sulfoxide was added to each well to solubilize the formazan crystals. The optical density was determined at 492 nm (Vero cells) and 570 nm (HepG2 cells; Stat Fax 2100 microplate reader, Awareness Technology, Inc, USA). The cell viability was expressed as a percentage of the control absorbance in the untreated cells after subtracting the appropriate background and the average cytotoxic concentration (CC_50_) was determined by linear regression. Samples with CC_50_ >200 μg/mL were considered of low cytotoxicity. Selectivity index (SI) for the anti-malarial activity was then calculated based on the rate between CC_50_ and IC_50_ for the in vitro activity against *P. falciparum* [28].

## Results

### Phytochemical studies

**Fig. 1:**
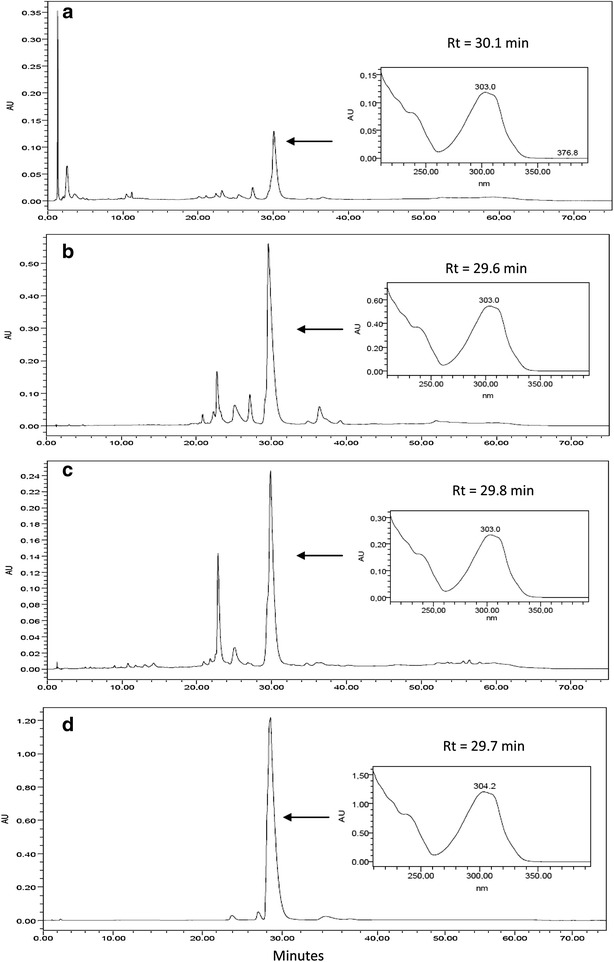
HPLC-DAD profiles of *Aspidosperma parvifolium* trunk bark ethanol extract and fractions. **a** Trunk bark ethanol extract (EEAP); **b** alkaloid fraction (EEAPA); **c** neutral fraction (EEAPN); **d** uleine

HPLC–DAD chromatograms showed a peak for the same major component in the ethanol extract (RT = 30.1 min; Fig. 1a), alkaloid fraction (RT = 29.6 min, Fig. 1b) and neutral fraction (RT = 29.8 min, Fig. 1c) that might correspond to uleine (Fig. 1d). The UV spectra are suggestive of indole alkaloid [29] (Fig. 1a–d).

**Fig. 2:**
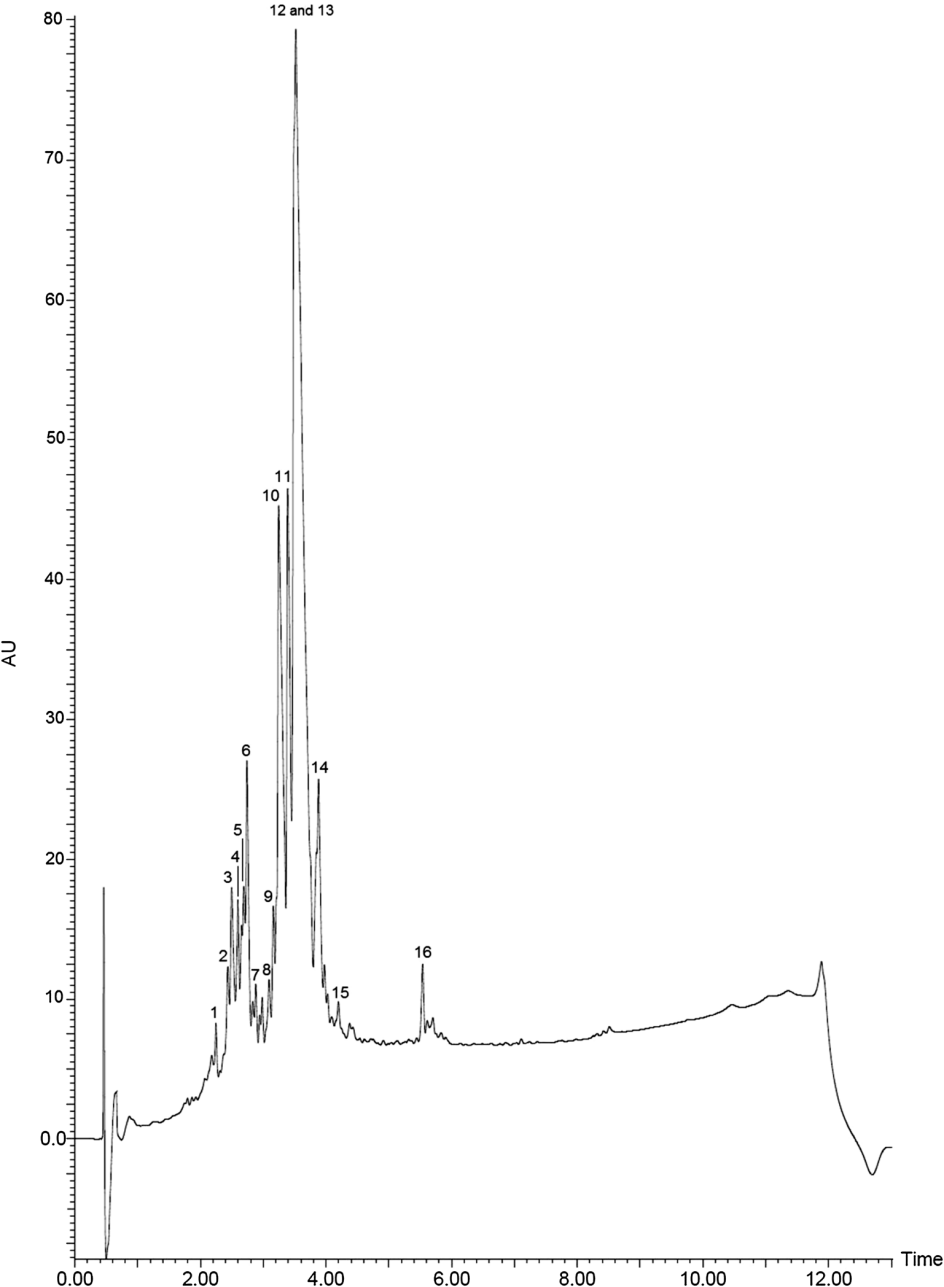
UPLC-DAD profile of *Aspidosperma parvifolium* trunk bark alkaloid fraction. (*1*) Spegazzinidine, (*2*) 1,13-Dihydro-13-hydroxyuleine, (*3*) aspidocarpine, (*4*) deacetylaspidospermine, (*5*) pyrifolidine, (*6*) 1-methyldeacetylaspidospermidine, (*7*) dasycarpidone, (*8*) aspidospermidin-19-ol, (*9*) N-acetylaspidospermidine, (*10*) *N*-demethyluleine, (*11*) apparicine, (*12*) uleine, (*13*) 3-*epi*-uleine, (*14*) aspidospermine, (*15*) dasycarpidol, (*16*) dasycarpidan

The on-line UPLC-DAD  chromatogram of the alkaloid fraction shows also a predominant peak (RT = 3.49 min) (Fig. 2) related to indole alkaloids (Fig. 2). This peak must correspond to the most intense one in Fig. 1a–c. The difference in retention times (RT) is a consequence of the different mobile phases used in the experiments as well as to the different equipments in each case, HPLC and UPLC.

**Fig. 3:**
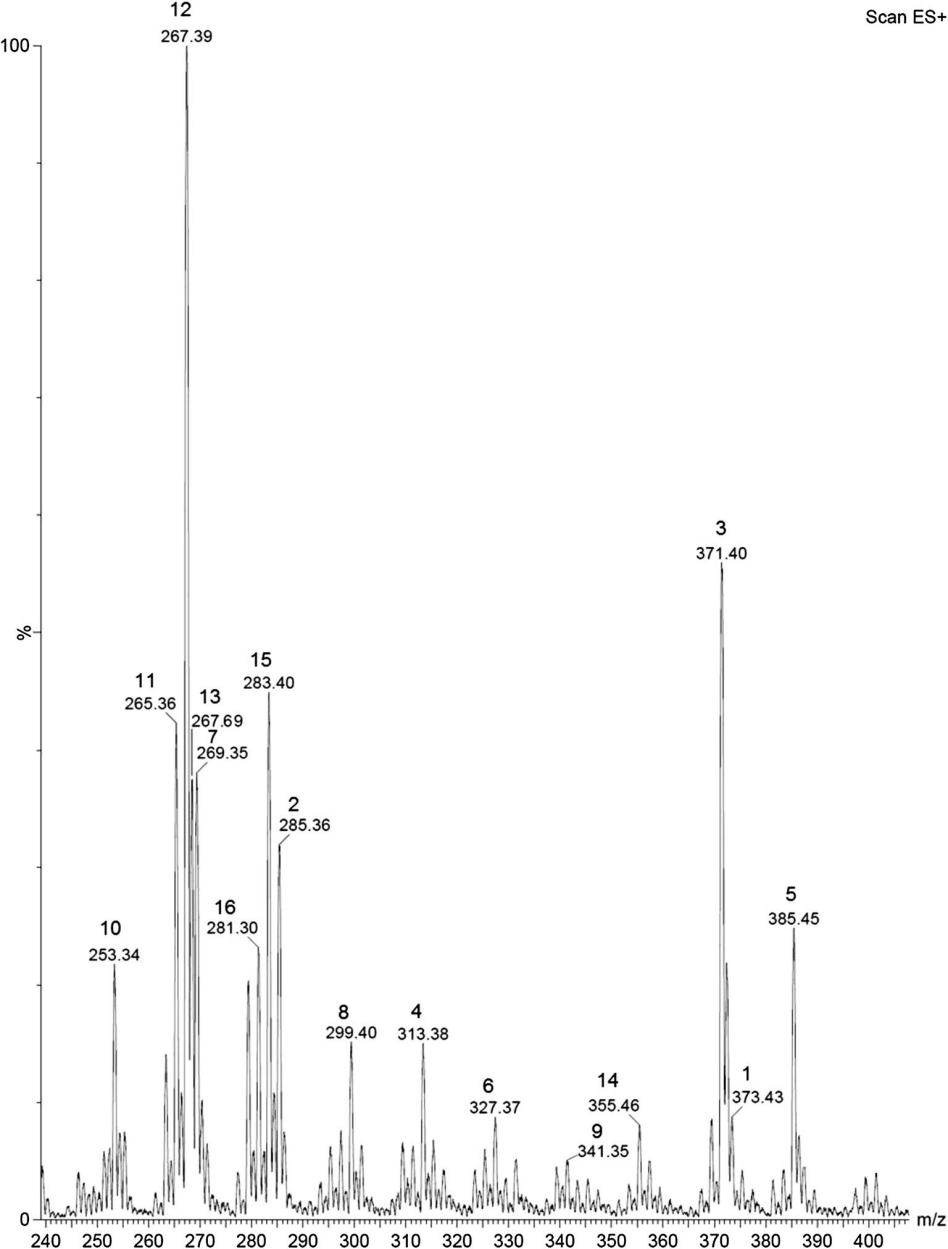
UPLC-ESI–MS spectrum of *Aspidosperma parvifolium* trunk bark alkaloid extract. (*1*) Spegazzinidine, (*2*) 1,13-dihydro-13-hydroxyuleine, (*3*) aspidocarpine, (*4*) deacetylaspidospermine, (*5*) pyrifolidine, (*6*) 1-methyldeacetylaspidospermidine, (*7*) dasycarpidone, (*8*) aspidospermidin-19-ol, (*9*) N-acetylaspidospermidine, (*10*) *N*-demethyluleine, (*11*) apparicine, (*12*) uleine, (*13*) 3-*epi*-uleine, (*14*) aspidospermine, (*15*) dasycarpidol, (*16*) dasycarpidan

**Fig. 4:**
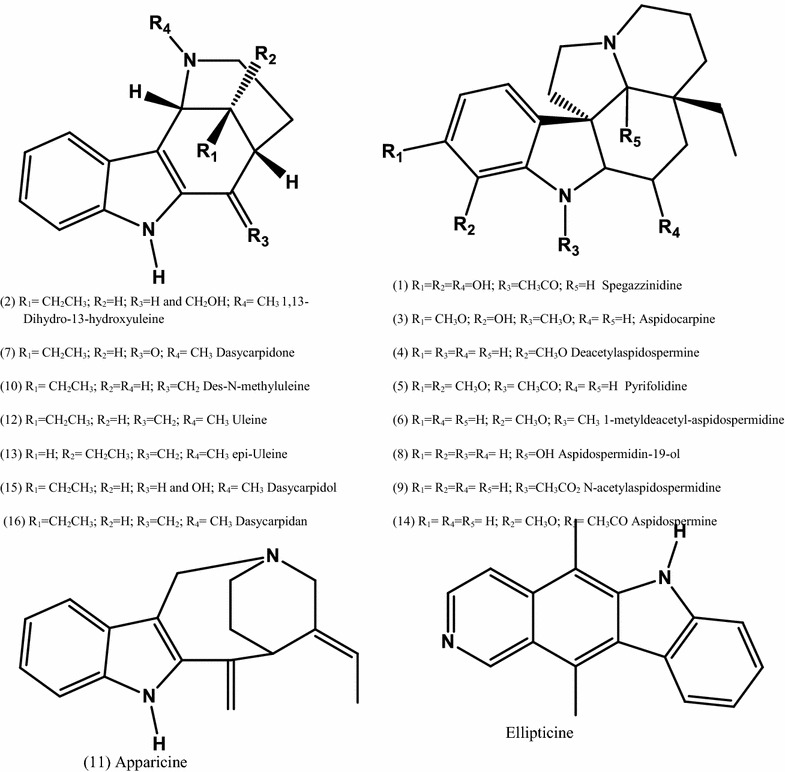
Chemical structures of the indole alkaloids identified in *Aspidosperma parvifolium* trunk bark alkaloid extract

The UPLC-ESI–MS spectrum of the alkaloid fraction disclosed distinct peaks for uleine and 3-*epi*-uleine, demonstrating that superposition of these alkaloids peaks was occurring in Figs. 1a–c and 2. This behaviour is a consequence of the close polarity of these two molecules that are just epimers at C-3 position and explains the difficulty for isolation of pure uleine although being the major alkaloid fraction constituent. Further analyses of the alkaloid fraction was performed by tandem mass spectrometry (UPLC-ESI–MS/MS) (Fig. 3), a variant of HPLC that affords significant advantages in resolution, speed, and sensitivity for analytical determinations, particularly when coupled to mass spectrometers capable of high-speed acquisitions allowing studies on the fragmentations of each component under electron spray conditions [30]. The observed mass fragments (Table 1) are consistent with the structural classes of these alkaloids that belong to the aspidospermane and plumerane types, [31–34] and full fragmentation mechanisms will be published elsewhere. Only six out of the 16 alkaloids identified have been previously isolated from *Aspidosperma parvifolium*, e.g., *N*-methyltetrahydroellipticine, uleine, 3-*epi*-uleine, *N*-demethyluleine, *N*-demethyl-dihydrouleine, and apparicine [15, 35] (Fig. 4).

**Table 1:** Alkaloids identified in *Aspidosperma parvifolium* trunk bark alkaloid extract by UPLC-DAD-ESI–MS/MS and HPLC-ESI-MICROTOF-MS

Compounds	Peak number	Rt (min) UPLC-ESI-MS	UV λ_max_ (nm) UPLC-DAD	[M+H]^+^ UPLC-ESI-MS (m/z)	HRMS HPLC-ESI- MICROTOF-MS (m/z)	UPLC-ESI-MS/MS fragments (m/z)
Spegazzinidine	1	2.06	317.13	373.43		373.11, 354.90, 341.10, 323.90, 311.42, 295.0, 279.10, 247.79, 231.91, 205.95, 183.71, 124.07, 108.86
1,13-Dihydro-13- hydroxyuleine	2	2.26	316.13	285.36	285.1949	285.11, 254.11, 238.10, 236.29, 210.16, 208.0, 206.01, 197.97, 195.15, 182.15, 168.15, 167.02, 155.96, 144.02, 130.02, 124.07, 121.89
Aspidocarpine	3	2.50	304.13	371.40	371.1975	371.22, 353.95, 339.21, 321.0, 270.22, 249.68, 245.90, 227.69, 220.13, 199.71, 173.75, 154.10, 152.08, 142.88, 107.97
Deacetylaspidospermine	4	2.60	317.13	313.38	313.1953	313.08, 251.89, 222.09, 206.08, 180.12, 168.03, 151.96, 140.05, 131.92
Pyrifolidine	5	2.62	317.13	385.45	–	385.14, 367.12, 353.07, 327.11, 309.09, 256.10, 231.97, 167.89, 121.07
1-Methyldeacetyl-aspidospermidine	6	2.88	310.13	327.37	327.2061	327.01, 309.16, 296.15, 283.14, 278.30, 252.21, 236.19, 197.97, 170.0, 168.24, 130.72, 129.21
Dasycarpidone	7	2.74	319.13	269.35	269.1628	238.16, 210.12, 209.04, 192.16, 182.11, 167.06, 130.01
Aspidospermidin-19-ol	8	3.25	302.13	299.40	299.1806	299.15, 252.02, 234.75, 233.75, 221.33, 208.10, 206.34, 196.07, 169.16, 159.39, 150.07, 138.03, 122.97, 109.17
N-Acetylaspidospermidine	9	3.27	303.13	341.35	341.1856	341.15, 322.96, 309.17, 304.58, 295.20, 294.07, 281.29, 266.8, 251.90, 236.22, 223.01, 183.73, 168.12, 121.86, 107.82
Des-N-methyluleine	10	3.35	304.13	253.34	253.1686	236.10, 221.10, 208.09, 207.16, 206.03, 194.09, 193.03, 182.03, 181.03, 167.02
Apparicine	11	3.39	303.13	265.36	265.1678	265.11, 250.02, 236.12, 222.16, 208.14, 207.20, 206.09, 194.12, 180.0, 158.03, 134.01, 108.17
Uleine	12	3.49	303.13	267.39	267.1845	236.10, 221.04, 208.09, 207.09, 206.16, 193.03, 182.09, 167.02
*Epi-*uleine	13	3.53	304.13	267.69	267.1853	236.16, 221.04, 208.09, 207.03, 206.03, 194.03, 193.03, 182.03, 181.03, 167.02
Aspidospermine	14	3.63	297.13	355.46	355.1993	355.14, 323.30, 313.08, 268.19, 256.22, 218.07, 173.81, 164.17, 124.01, 122.05
Dasycarpidol	15	3.88	304.13	283.40	283.1793	236.23, 221.04, 208.09, 207.09, 206.16, 193.09, 192.15, 181.50, 167.0
Dasycarpidan	16	5.51	303.13	281.30	-	281.05, 236.07, 222.08, 194.04

(−) species not detected

*Rt* retention time, *UV* ultraviolet, *UPLC-DAD-ESI–MS* ultra performance liquid chromatography photo diode array electron spray mass spectrometry, *HRMS* high resolution mass spectrometry, *HPLC-ESI-MICROTOF-MS* high performance liquid chromatography electro spray ionization time-of-flight mass spectrometry

The chemical structure of uleine was characterized by spectroscopic data (UV, IR, MS and NMR) in comparison with literature data [15, 28, 36] and its identification was confirmed by direct comparison with authentic sample that was previously isolated at UFMG [15].

### Anti-malarial activity

The ethanol extract of *Aspidosperma parvifolium* trunk bark disclosed a good in vitro activity against *P. falciparum* W2 strain by the HRPII assay (IC_50_ = 10.11 ± 2.22 µg/mL) and a moderate activity by the microscopic method against parasite W2 and 3D7 strain (IC_50_ = 32.75 ± 1.06 and 20.51 ± 0.70 µg/mL, respectively). Higher IC_50_ values were determined by the [^3^H]-hypoxanthine method against W2 and 3D7 strains (IC_50_ = 42.51 ± 6.33 and 38.20 ± 0.33 µg/mL, respectively) (Table 2). The neutral fraction was active against parasite W2 strain by the HRPII method (IC_50_ = 9.71 µg/mL) and its IC_50_ was close to that one of the ethanol extract (IC_50_ = 10.11 ± 2.22 µg/mL) against the same parasite strain. The alkaloid fraction and uleine were very active disclosing close IC_50_ values against the parasite W2 strain by the three methods, the lowest value being determined by the microscopic method: 0.98 ± 0.20 and 0.75 ± 0.10 µg/mL, respectively. Higher IC_50_ values were determined for all the samples against W2 parasite by the radioisotopic method than by the microscopic method and it was approximately ten-fold higher for the alkaloid fraction and uleine. For the purpose of comparison, the data on the anti-malarial activity of apparicine, aspidocarpine, and ellipticine, the only previously assayed alkaloids [37, 38] out of the 16 here identified in *Aspidosperma parvifolium*, are included in Table 2.

**Table 2:** Antiplasmodial activity of ethanol bark extract of *Aspidosperma parvifolium,* fractions and uleine along with literature data for apparicine, aspidocarpine, and ellipticine

Extract/fractions/compounds	IC_50_ (µg/mL) *P. falciparum* W2			IC_50_ (µg/mL) *P. falciparum* 3D7	
	MM	HM	HRPII	MM	HM
Choroquine	0.02 ± 0.002	0.018 ± 0.0005	0,0856 ± 0.0201	0.0013 ± 0.0001	0.002 ± 0.0004
Mefloquine	0.016 ± 0.002	0.044 ± 0.008	ND	0.048 ± 0.0007	0.052 ± 0.0006
EEAP	32.75 ± 1.06	42.51 ± 6.33	10,11 ± 2,22	20.51 ± 0.70	38.20 ± 0.33
EEAPA	0.98 ± 0.20	8.0 ± 1.19	0.43 ± 0.11	7.63 ± 0.31	43.05 ± 4.30
EEAPN	15.02 ± 2.83	38.02 ± 3.76	9,71 ± 2,63	17.75 ± 0.35	18.2 ± 1.75
Uleine	0.75 ± 0.10	8.78^a^	2.95 ± 1.47	11.90 ± 0.10	ND
Apparicine^b^		3.0 ± 1.4	3.2 ± 2.7		
Aspidocarpine^b^		5.4 ± 2.5	4.4 ± 0.8		
Ellipticine^c^	0.018				

*EEAP* ethanol extract of *Aspidosperma* *parvifolium* trunk bark, *EEAPA* alkaloid fraction prepared from EEAP, *EEAPN* neutral fraction prepared from EEAP, *MM* microscopic method, *HM* [^3^H]-hypoxanthine method, *ND* not determined

^a^one triplicate experiment

^b^Ref. [36] *P. falciparum* W2

^c^Ref. [35] *P. falciparum* K1

Differences in the IC_50_ values for the microscopic method and the radioisotopic assay might be related to the time of incubation of the parasites with the samples: 72 and 42 h, respectively. However, for the radioisotopic assay and the HRPII method that has had similar incubation times, an important factor that might explain these differences, might be the intrinsic sensitivity of each technique. Interestingly, higher potency was generally observed against parasite W2 strain, which is chloroquine resistant, than against the 3D7 strain, a chloroquine-sensitive *P. falciparum* strain, except for the ethanol extract.

The relatively close IC_50_ values for the neutral fraction and the crude ethanol extract (Table 2) could be explained by the fact that alkaloids are still present in the neutral fraction indicating that the acid–base process used for extraction of alkaloids from the ethanol extract was not complete, as is shown by the HPLC–DAD chromatogram (Fig. 1c).

It is interesting to call attention for the fact that, only three (apparicine, aspidocarpine and ellipticine) out of the 16 identified alkaloids in the trunk bark of *Aspidosperma parvifolium* were previously evaluated against *P. falciparum* [37, 38]. Ellipticine is the most active alkaloid amongst those shown in Table 2 but it is highly cytotoxic while uleine was not active against a panel of tumoral cells [18].

Uleine is a monoterpenoid indole alkaloid and its chemical structure can be related to those of quinine and synthetic quinoline anti-malarials, such as chloroquine, with the nitrogen of the indole unit corresponding to that of the quinoline system. These nitrogens are of low basicity. A second nitrogen, of a tertiary amine, and therefore of higher basicity, is present in both the systems. The quinoline system is the pharmacophore that plays a crucial role in the complexation to ferriprotoporphyrin IX (FPIX) resulting in inhibition of haemozoin formation and parasite growth. On the other hand, the presence of a basic amino group in the side chain is generally considered essential for trapping high concentrations of the drug in the acidic digestive vacuole of the parasite. It is known that these structural characteristics are important for the inhibition of the haem polymerization, the mechanism of action of quinolines as anti-malarials [39] and, possibly, of the monoterpenoid indole alkaloids, as well.

Recently, uleine was isolated from *Aspidosperma olivaceum* and its anti-malarial activity was also evaluated against the chloroquine-resistant strain W2 by the HRPII (IC_50_ = 3.2 ± 1.8 mg/mL) and the [^3^H]-hypoxanthine (IC_50_ = 7.0 ± 0.0 mg/mL) assays [38]. These data confirm the results reported here and point to uleine as a novel anti- malarial hit.

### Citotoxicity and selectivity index (SI)

The ethanol extract was not cytotoxic to Vero and HepG2 cell lines up to the concentrations of 500 and 1000 mg/mL, respectively. Fractionation of ethanol extract led to fractions of higher cytotoxicity (Table 3). The neutral fraction showed low cytotoxicity against both Hep G2A16 and Vero cell lines (CC_50_ = 251.0 and 449.3 μg/mL, respectively). The alkaloid fraction was more cytotoxic to HepG2 (CC_50_ 74.4 ± 15.5 µg/mL) than to Vero cells (CC_50_ 299.7 µg/mL) (Table 3). On the other hand, chromatographic fractionation of the alkaloid extract led to uleine that showed low cytotoxicity against both Hep G2A16 and Vero cell lines (CC_50_ = 374.6 and 301.2 μg/mL, respectively). The low cytotoxicity of uleine against 65 human cancer cell lines was previously reported [18]. Surprisingly, a higher cytotoxicity of uleine (CC_50_ = 52 ± 10.0 μg/mL) to HepG2 cell line was recently assigned [38]. The extract, neutral fraction and uleine disclosed high selectivity indexes (SI > 10) (Table 3) while lower selectivity was determined for the 3D7 chloroquine-sensitive strain of *P. falciparum* (SI < 10) (Table 3). In summary, uleine disclosed high SI and greater selectivity for the resistant strain W2, than the ethanol extract and the alkaloid fraction (Table 3).

**Table 3:** Cytotoxicity of ethanol extract trunk bark of *Aspidosperma* *parvifolium*, derived fractions and uleine to Vero and HepG2 cells

Extract/fractions/compounds	Vero cells			HepG2 cells		
	CC_50_ (µg/mL)	SI W2	SI 3D7	CC_50_ (µg/mL)	SI W2	SI 3D7
EEAP	>500	>15.3	>24.4	>1000	30.5	48.7
EEAPA	299.7	305.8	39.3	74.4	75.9	9.7
EEAPN	449.3	29.9	25.3	251.0	16.7	14.1
Uleine	374.6	499.5	31.5	301.2	407.6	25.3
Chloroquine	ND	ND	Nd	185.0	9250.0	142,307.7

*EEAP* ethanol extract of *Aspidosperma* *parvifolium* trunk bark, *EEAPA* alkaloid fraction prepared from EEAP, *EEAPN* neutral fraction prepared from EEAP, *SI* selectivity index

When relating anti-malarial activity and cytotoxicity, it is noted that the fractionation performed was successful. Although no significant difference is observed between the IC_50_ values determined for uleine and the alkaloid fraction (Table 2), they are however quite distinct with respect to the cytotoxicity (CC_50_), uleine being safer (less cytotoxic) (Table 3).

## Conclusion

Ethanol extract of *Aspidosperma parvifolium* trunk bark afforded uleine that discloses high in vitro anti-malarial activity and selectivity against the chloroquine-resistant *P. falciparum* strain W2. Besides, the alkaloid fraction showed anti-malarial activity similar to uleine. However, uleine showed lower cytotoxicity and increased SI when compared to the alkaloid fraction. Uleine was demonstrated to be the major constituent in the alkaloid fraction of *Aspidosperma parvifolium* trunk bark and 15 other indole alkaloids have been identified along with uleine. The high chemical diversity of alkaloids from *Aspidosperma* species and the small number of these alkaloids that have been assayed for anti-malarial activity coupled to the traditional use of several species of this taxon to treat malaria in Brazil, as well as in other meso- and South American countries, make further investigations of plants in this genus of great interest in the quest for natural anti-malarial drugs [12].
